# Olanzapine induced autophagy through suppression of NF‐κB activation in human glioma cells

**DOI:** 10.1111/cns.13127

**Published:** 2019-04-07

**Authors:** Ying Zhu, Yi‐Fan Zhao, Rui‐Si Liu, Ya‐Jie Xiong, Xiao Shen, Yan Wang, Zhong‐Qin Liang

**Affiliations:** ^1^ Department of Pharmacology, College of Pharmaceutical Sciences Soochow University Suzhou China

**Keywords:** autophagy, glioma, MGMT, NF‐κB, olanzapine

## Abstract

**Aims:**

Our laboratory previously reported that olanzapine treatment inhibited growth of glioma cell lines and hypothesized that autophagy may be involved in the proliferation inhibitory effects of olanzapine. However, the mechanisms of olanzapine‐contributed autophagy activation are unclear.

**Methods:**

The inhibitory effects of olanzapine on glioma cells were evaluated by CCK8 assay, Hoechst 33258 staining and annexin V‐FITC/PI staining. Western blotting, nuclear separation techniques, and immunofluorescence assays were used to investigate the relationship between the inhibition of NF‐κB and autophagy activation by olanzapine.

**Results:**

In this work, we verified that olanzapine increased autophagic flux and autophagic vesicles. In addition, we confirmed that autophagy was related to NF‐κB inhibition in cancer progression, especially with the nuclear translocation of p65. Furthermore, we demonstrated that autophagy induced by olanzapine could be impaired with TNFα cotreatment. We also found that olanzapine had an inhibitory effect on T98 cells with positive MGMT protein expression, which may involve the inhibition of MGMT through effects on NF‐κB.

**Conclusions:**

Our findings identify a pathway by which olanzapine induces autophagy by depressing NF‐κB in a glioma cell line, providing evidence which supports the use of olanzapine as a potential anticancer drug.

## 1 INTRODUCTION

Malignant glioma is the prevalent type of brain tumor in humans.^1^ Although there are many treatments, including chemotherapy, surgery, and radiation, the survival rate is still low. The majority of glioblastoma patients survive <12 months even with aggressive treatment.^2^ Temozolomide (TMZ) is a standard chemotherapeutic drug for the treatment of the glioblastoma.^3^ However, resistance to TMZ is an obstacle to effective treatment. It is reported that the main mechanism of glioma cell resistant to TMZ is linked to the expression of the O^6^‐methylguanine DNA methyltransferase (MGMT) gene.^4^ Thus, it is necessary to identify an effective antiglioblastoma drug for brain cancer patients, especially for those resistant to TMZ.

Olanzapine is an atypical antipsychotic drug, and some reports suggest it may inhibit the proliferation of tumor cells. In recent years, olanzapine has been frequently used as a treatment for nausea and vomiting in cancer patients because of its known side effect (increased appetite).^5^ Our previous experiments indicated that olanzapine could inhibit tumor proliferation both in vivo and in vitro.^6^ The current study verifies that this inhibitory effect is related to autophagy.

Autophagy is a self‐digestion process that deactivates damaged organelles and aggregated proteins to promote self‐balance.^7^ Autophagy can promote cell survival or cell death via different mechanisms.^8^, ^9^ We have found that the activation of autophagy by olanzapine contributes to cell death in glioma cells. However, the mechanism of olanzapine‐induced autophagy is not understood. Nicolau‐Galmes et al reported that H1‐antihistamine induces autophagy; therefore, as a histamine H1 receptor antagonist,^10^ olanzapine is supposed to induce autophagy. In addition, some studies report that olanzapine has the ability to enhance p‐AMPK activation.^11^, ^12^ Once p‐AMPK is activated, autophagy is stimulated, leading to glioma related cell death.^13^ The AMPK/mTOR pathway is associated with NF‐κB.^14^

As an important nuclear transcription factor related to tumor growth, NF‐κB is overexpressed and activated in malignant glioma cells, thus promoting tumor cell survival.^15^, ^16^ The NF‐κB family consists of three subunits, including p50, p65, and IκBα. Typically, in nonstimulated cells, NF‐κB is present in the cytoplasm and bound to IκBα to maintain transcriptional inactivation. Some outside stimuli, such as the tumor necrosis factor‐alpha (TNFα), can induce phosphorylation and ubiquitination of upstream IκBα, leading to its degradation, thereby releasing NF‐κB and promoting its activation and nuclear translocation.^17^ When NF‐κB translocates to the nucleus, it increases the expression of target genes associated with inflammation and cell cycle regulation.^18^, ^19^ In some types of cancer cells, constitutive activation of NF‐κB decreases cell sensitivity to cancer treatment.^20^ At the same time, anticancer treatments enhance NF‐κB activity, leading to drug resistance and treatment failure.^21^

The relationship between NF‐κB and autophagy is complex, as the AMPK/mTOR pathway often interacts with NF‐κB.^14^ Some studies report that NF‐κB can activate the autophagy inhibitor mTOR and thus contribute to good effects on autophagy inhibition.^22^ On the contrary, some recent research suggests that autophagy could also induce NF‐κB inactivation by degrading its upstream IκB kinase (IKK).^23^ In general, NF‐κB increases tumorigenicity and has inhibitory effects on autophagy in cancer.^24^ In this study, we demonstrated that olanzapine activated autophagy through the inhibition of NF‐κB, ultimately promoting autophagy and apoptosis.^6^

NF‐κB is implicated in the regulation of MGMT, and DNA injury often activates both MGMT and NF‐κB.^25^ Lavon et al reported that the p65 homodimer played a main role in NF‐κB‐induced MGMT expression. They also confirmed that NF‐κB‐binding sites were present in the promoter region of MGMT and that MGMT had specific and direct interactions with NF‐κB.^26^ In the present study, we demonstrated that olanzapine could activate autophagy by interfering with the nuclear translocation of NF‐κB. We also demonstrated that olanzapine could inhibit MGMT by blocking NF‐κB. This novel pathway may provide a foundation for the development of olanzapine as an antitumor intervention.

## 2 MATERIAL AND METHODS

### 2.1 Reagents

Olanzapine was purchased from Selleckchem (USA) and stored in −30°C (50 mM in DMSO). NE‐PER Nuclear and Cytoplasmic Extraction Reagents were bought from Thermo Scientific (Rockford, USA). LC3 antibody was purchased from Abcam (Inc, Cambridge, MA, USA). P62 and MGMT antibodies were obtained from Santa Cruz (CA, USA). Antibodies for Bcl‐2, Bax, NF‐κB p65, IκBa, GAPDH, lamin B were acquired from Cell Signaling Technology (Beverly, MA). TNFa and SN50 were purchased from Med Chem Express (Monmouth Junction, NJ, USA).

### 2.2 Cell culture

Glioma cell lines T98, LN229, and U87 were all purchased from American Type Culture Collection (ATCC). All cells were cultured in 10% FBS/DMEM and incubated at 37°C in a humidified atmosphere of 5% CO_2_.

### 2.3 Cell viability assay

Cell viability was measured by CCK8 assay. T98, LN229, or U87 cells were grown in 96‐well plates and incubated overnight. Then, different concentrations of olanzapine were added for 24, 48, and 72 h before 10 μL CCK8 solution. The absorbance of each well was acquired by using an automatic fluorescence microplate reader with a wavelength of 450 nm after 1‐4 hours. The experiments were repeated for 3 times with each control group, and experimental group was performed in six wells. The cell viability results were as follows: cell viability % = (OD_treat_ − OD_blank_)/(OD_con_ − OD_blank_) × 100.

### 2.4 Western blot

Cells were collected and washed with PBS followed by cracking with cold lysis buffer containing protease inhibitor cocktail (Roche, Mannheim, Germany). The supernatant of the protein was extracted by centrifugation at 12 000 rpm for 15 min, and protein concentration was measured by BCA Protein Assay Kit (Thermo, Rockford, IL, USA). Equal amounts of proteins of each sample were separated on 12% sodium dodecyl sulfate‐polyacrylamide gel electrophoresis (SDS‐PAGE) and then transferred onto a polyvinylidene difluoride membranes. The membranes were first blocked with 5% nonfat milk at room temperature and then incubated with the primary antibodies at 4°C overnight in TBST. The membranes were washed next day and incubated with secondary antibodies for 1 hour. Odyssey Infrared Imaging System was used to detect the blots, and Image J software was used to quantify the data.

### 2.5 Hoechst 33258 staining

DNA dye Hoechst 33258 was used to detect apoptotic cells. The cells were treated with drugs separately, fixed with methanol for 15 minutes at 4°C, and then incubated with Hoechst 33258 at room temperature for 5 minutes. After washed with PBS, apoptotic cells were attached to glass slides and observed under fluorescence microscope. The images were taken by a digital camera. Cells with abnormal nuclei (crenation, condensation, and fractionation) were labeled as apoptotic cells.

### 2.6 Cell death detected by flow cytometry

Cellular death was assessed by Annexin V‐FITC/PI apoptosis detection kit (Beyotime, Jiangsu, China) as per the instructions. Cells were collected 48 hours after treatment and resuspended with 200 μL binding buffer, and then incubated with 5 μL Annexin V‐FITC and 10 μL propidium iodide (PI) at room temperature for 15 minutes in the dark. A total of 10 000 cells were collected from each tube for data collection, and cell mortality was analyzed by LSRII flow cytometry and FACSDiva software.

### 2.7 NE‐PER nuclear and cytoplasmic extraction

After treatment, the cells were collected and washed with PBS, and resuspended in CER I (cytoplasmic extraction reagent I) according to the volume of cell precipitation, and incubated on ice for 10 minutes after severe oscillation, and then added cold CER II (cytoplasmic extraction reagent II) and continued to incubate on ice for 1 minute, centrifugated at 16 000 g for 5 minutes at 4°C. The supernatant was collected as cytoplasmic extract, and the insoluble precipitate (containing nuclei) was suspended in cold NER (nuclear extraction reagent), agitated for 40 minutes on the ice, and centrifugated at 16 000 g for 10 minutes at 4°C. Supernatant was collected and used as the nuclear extract and stored at −80°C for analysis.

### 2.8 Immunofluorescence staining

T98 cells were plated on cover slips. After specific treatments for 48 hours, cells were fixed in 4% paraformaldehyde for 10 minutes at 4°C, washed with PBS and permeabilized with 0.1% Triton X‐100 for 5 minutes, and blocked in 1% BSA and 0.1% Triton X‐100. The cells were then incubated with primary antibodies at 4°C overnight. The next day, cells were incubated with rabbit Alex Fluor 488 (green) (1:200, Molecular Probes, Eugene) for 1 hour. After washing with PBS, cells were counterstained with DAPI for 10 minutes. Coverslips were examined by confocal microscope (Carl Zeiss, Jena, Germany).

### 2.9 Colony formation assay

A total of 500 cells were sown in 6‐well plates overnight and then treated with different concentrations of olanzapine. After 14 days of cell culture, crystal violet staining solution (Beyotime) was used to stain the cells. Number of visible colonies (>50 cells) was counted by microscope, and typical images were taken by a digital camera.

### 2.10 Statistical analyses

Data in the figures are expressed as mean ± SEM. All experimental data were repeated more than three times. One‐way ANOVA and Student's *t* test were used for statistical analysis. *P* < 0.05 denoted the difference was statistically significant.

## 3 RESULTS

### 3.1 Olanzapine inhibited growth and induced autophagy in human glioma cells

**Figure 1:**
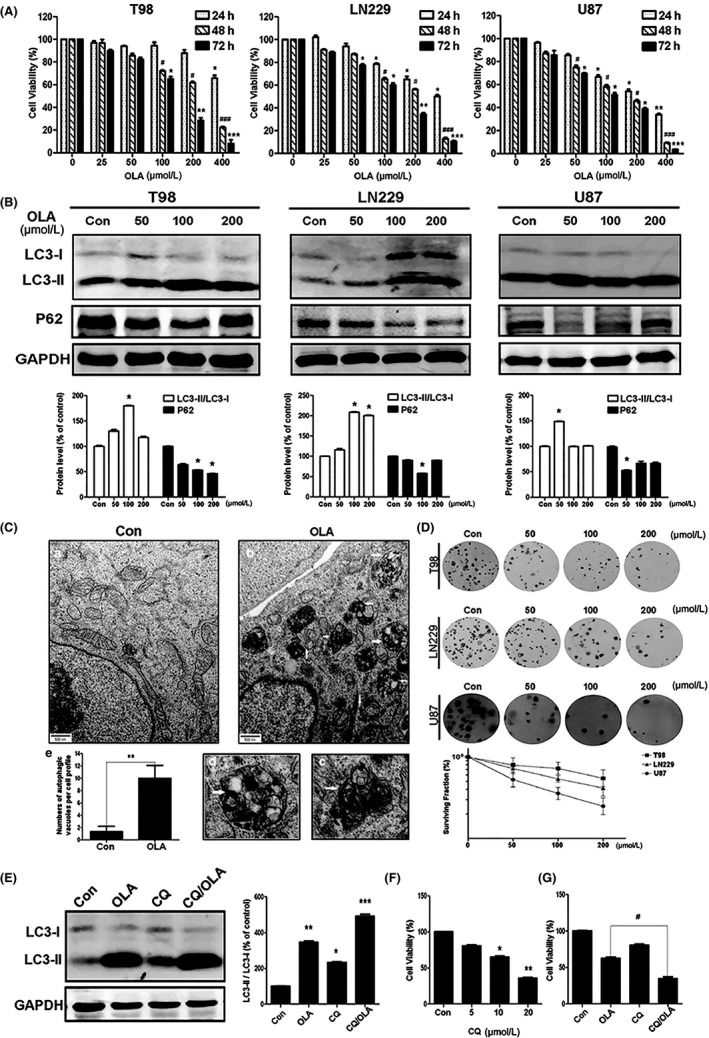
Olanzapine activated autophagy in glioma cells. A, T98, LN229, and U87 cells were treated with olanzapine (OLA) at different concentrations for 24, 48, or 72 h, respectively. Cell viability was determined by CCK8 assays. B, The expression of LC3 and p62 with OLA treatment (48 h) was evaluated by Western blotting in T98, LN229, and U87 cells. The GAPDH was used as a loading control (*P < 0.05 vs control). C, Representative electron micrograph of T98 cells are shown in Figure (a, b) with OLA treatment (100 μM, 48 h). The arrowheads indicated the presence of autophagic vacuoles. At higher magnification, autophagosomes‐containing mitochondria (c) or lysosomes (d) were found in cells treated with OLA. (e) The number of autophagic vacuoles was determined in each cell (**P < 0.01 vs control). D, Colony formation assays. E, T98 cells were incubated in 100 μM OLA for 48 h with or without CQ (5 μM, 30 min) pretreatment. Cell extracts were analyzed for LC3‐II with specific antibodies (*P < 0.05, **P < 0.01, ***P < 0.001 vs control). F, Cell viability was determined by CCK8 assays with CQ treatment for 48 h in T98 cells (*P < 0.05, **P < 0.01 vs control). G, T98 cells were incubated in 100 μM OLA for 48 h with or without CQ (5 μM, 30 min) pretreatment, and then, CCK8 was used to detect cell viability (^#^ *P* < 0.05 vs OLA group)

The effects of olanzapine on glioma cells were assessed with the CCK8 assay. We treated T98, LN229, and U87 cells with 0‐400 µΜ olanzapine for 24, 48, 72 hours, respectively, and evaluated the cell viability of these three glioma cell lines. As shown in Figure 1A, olanzapine had significant inhibitory effects in a dose‐ and time‐dependent manner, indicating that olanzapine has robust inhibitory effects on the growth of the glioma cell lines.

Early reports from our laboratory indicated that the antitumor effects of olanzapine were associated with the activation of autophagy. Therefore, we used Western blotting analysis to measure the expression levels of autophagy‐related LC3 and p62 proteins. During the formation of the autophagosome, the microtubule‐associated protein light chain 3 (LC3‐I) is converted to its lipidized and membrane‐bound form, LC3‐II.^27^ As we expected, a significant increase of LC3‐positive spots was observed in olanzapine‐treated cells, indicating the activation of autophagosomes (Figure 1B). P62 (another marker of autophagy) binds to ubiquitinated targets and then transports their aggregates to the autophagosome for degradation by interacting with LC3‐II. Contrary to the increase of LC3‐II, the p62 protein, which acts as an autophagy substrate, was decreased due to degradation after olanzapine treatment (Figure 1B). We then conducted transmission electron microscopy experiments to observe and quantify autophagic vacuoles. We found that compared to untreated cells, the number of autophagy vacuoles in cells treated with olanzapine was increased, with significant lysosomes activation (Figure 1C). This may be related to the fusion of autophagosomes and lysosomes to form autophagic lysosomes and associated content degradation.

Furthermore, it was observed that chloroquine (CQ), a lysosomal inhibitor, had a cytotoxic effect on glioma cells (Figure 1F). However, when we cotreated with 100 µM olanzapine and 5 µM CQ (less toxic), the toxicity of olanzapine on T98 cells was significantly enhanced (Figure 1G). Moreover, compared with either olanzapine or CQ alone, the LC3‐II form was upregulated by the combined treatment, indicating that olanzapine may activate autophagic flux and induce apoptosis by accumulating autophagosomes (Figure 1E).

Under various stressful conditions, autophagy may mediate either tumor protection or tumor suppression. Based on the CCK8 assay data, cell viability was reduced by olanzapine treatment. Accordingly, when combined with the autophagy inhibitor 3‐MA, the reduced cell viability was prevented,^6^ suggesting that olanzapine induced autophagy contributed to tumor cell death in glioma cells. Colony formation assays were also used to confirm the suppressive effects of olanzapine. Survival rates were significantly decreased in a dose‐dependent manner with olanzapine treatment in T98, LN229, and U87 cells (Figure 1D). These results provided additional evidence that olanzapine‐induced autophagy was more likely to have antiproliferative effects on glioma cells.

**Figure 2:**
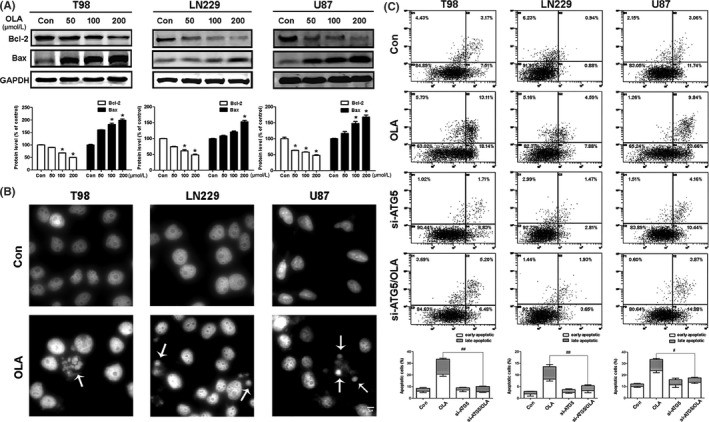
Olanzapine activated apoptosis in glioma cells. A, Total cell lysates were subjected to Western blot analysis to determine the protein levels of Bcl‐2 and Bax in T98, LN229, and U87 cells (*P < 0.05 vs control). B, Cells were pretreated with 100 μM OLA for 72 h, then subjected to Hoechst 33258 staining. Abnormal nuclei (condensation and fractionation) were determined by fluorescence microscopy (400×). C, Cells were treated with 100 μM OLA for 72 h, and then, Annexin V‐FITC/PI staining was used to determine the apoptosis in glioma cells (^#^ *P* < 0.05, ^##^ *P* < 0.01 vs OLA group)

Autophagy and apoptosis are different cell death pathways, but one often correlates with another. For example, large amounts of autophagosome aggregation can trigger apoptosis. In the current study, we observed that glioma cells produce large amounts of autophagosomes when treated with olanzapine, and hypothesized that olanzapine treatment may also induce apoptosis. We assessed protein levels of Bcl‐2 and Bax after treatment with olanzapine for 72 hours (Figure 2A). The decreased levels of Bcl‐2 and increased levels of Bax indicated that olanzapine induced apoptosis in glioma cells. Hoechst 33258 staining also revealed that when glioma cells were exposed to olanzapine for 72 hours, the morphology of the cells was altered, including nuclear rupture (Figure 2B). We also used annexin V‐FITC/PI to quantify the number of apoptotic cells and found that the rate of apoptosis increased with olanzapine treatment (Figure 2C). Further, by inhibiting ATG5 (autophagy gene) expression, olanzapine‐induced apoptosis could be abolished (Figure 2C). These results indicate that olanzapine treatment causes a concurrent increase in apoptosis and indicate that apoptosis is induced following autophagy, which was apparent at 48 hours. In addition, the inhibition of autophagy leads to a decrease of apoptosis.

### 3.2 Olanzapine suppressed the nuclear translocation of NF‐κB

**Figure 3:**
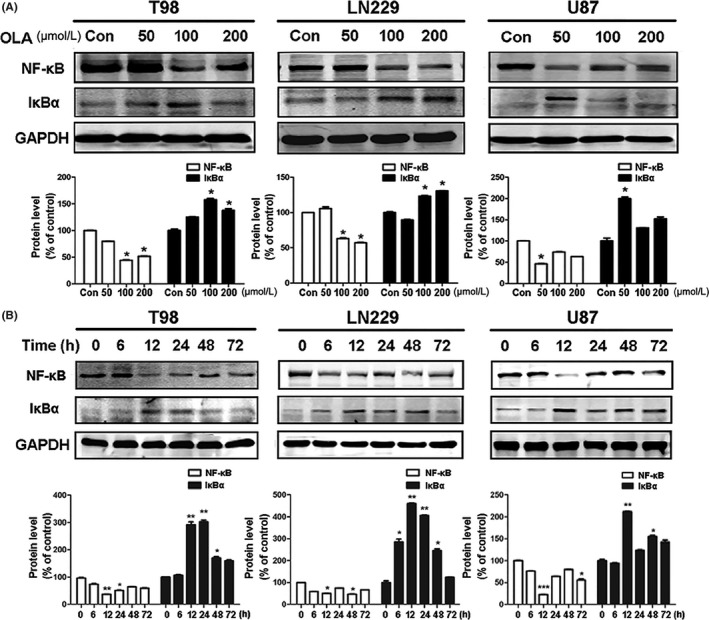
Olanzapine inhibited NF‐κB. A, The expression of NF‐κB and IκBa was evaluated by Western blotting after treated with OLA for 48 h in three cells. The GAPDH was used as a loading control (*P < 0.05, **P < 0.01, ***P < 0.001 vs control). B, Cells were treated with OLA for the indicated times before cell lysis. Cell extracts were analyzed by Western blotting using antibodies against NF‐κB (p65) and IκBa. The GAPDH was used as a loading control (*P < 0.05, **P < 0.01, ***P < 0.001 vs control)

Many studies have reported that IκBα plays an important role in the activation of NF‐κB to facilitate translocation of the latter into the nucleus and the subsequent regulation of target genes.^28^ To investigate the effects of olanzapine on NF‐κB activity, we first measured the levels of IκBα in cells treated with different concentrations of olanzapine for 48 hours. As shown in Figure 3A, olanzapine treatment significantly increased IκBα. Consistent with this observation, NF‐κB protein level was reduced in cells challenged with olanzapine, suggesting that olanzapine inhibited NF‐κB activation in glioma cells. Also, an increase of IκBα protein and a decrease of NF‐κB were detectable as early as 6 hours following olanzapine treatment, and the lowest level of NF‐κB protein was found at 12 hours (Figure 3B).

**Figure 4:**
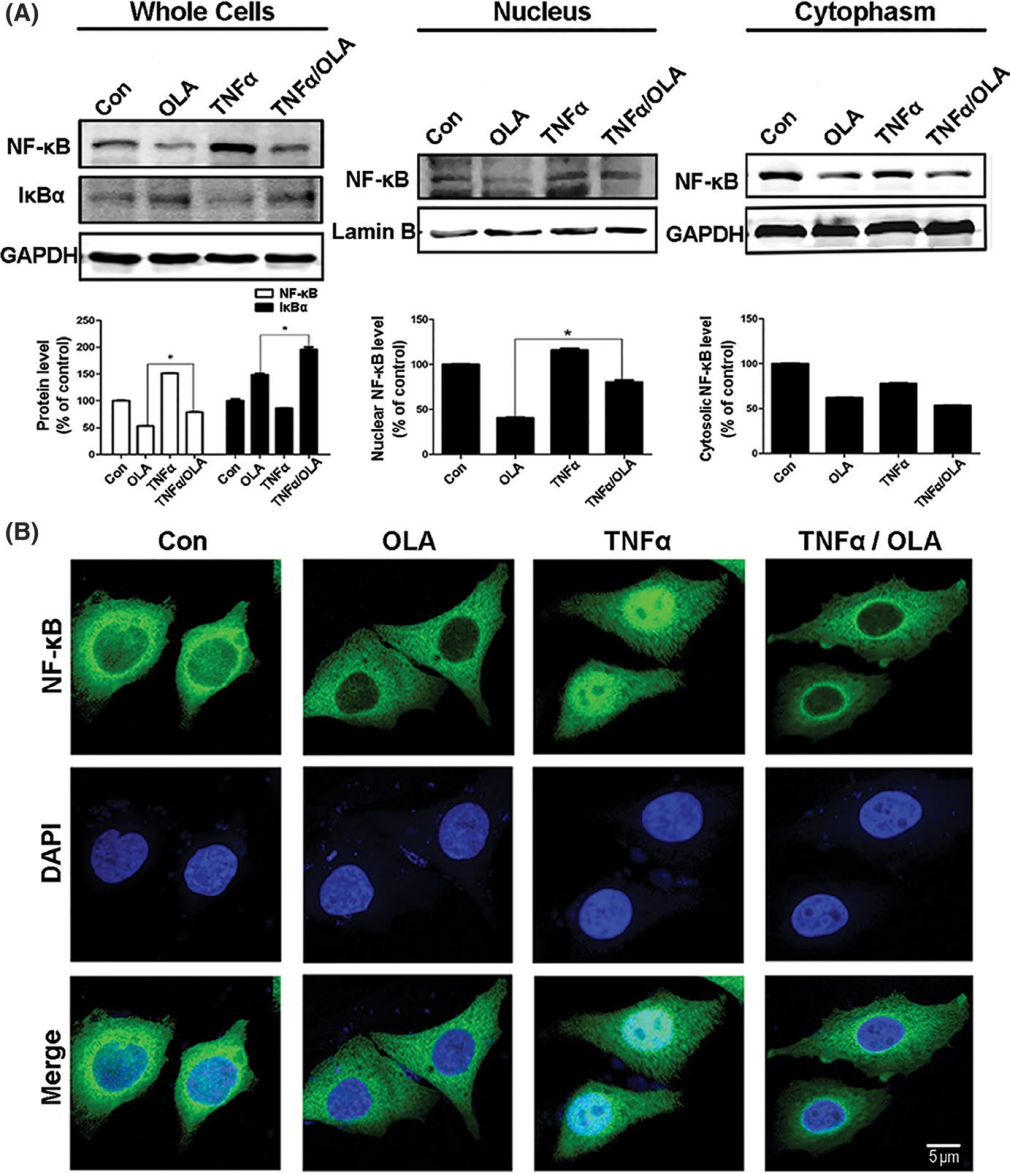
Olanzapine inhibited the nuclear translocation of NF‐κB. A, T98 cells were incubated in OLA (100 μM) for 48 h with or without TNFa (20 ng/mL, 30 min) pretreatment. (Left) Cellular extracts were prepared and analyzed by Western blotting using antibodies against NF‐κB (p65), IκBa, and GAPDH. The presence of NF‐κB in the cytosolic and nuclear extracts was also determined by Western blotting. GAPDH and lamin B were used as loading controls for the cytosolic and nuclear fractions, respectively (Middle, Right) (*P < 0.05 vs OLA group). B, Representative images of NF‐κB immunostaining were taken by confocal microscopy after OLA treatment (100 μM, 48 h) in T98 cells [Colour figure can be viewed at wileyonlinelibrary.com]

Because NF‐κB activation requires the translocation of its subunit RelA/p65 into the nucleus, we investigated the redistribution of p65 in the cytoplasm and nucleus after olanzapine treatment. Olanzapine treatment downregulated the p65 protein level in the cytosol and nucleus, with a robust reduction of the nuclear p65 protein level in T98 cells (Figure 4A). To verify these findings, we performed immunofluorescence microscopy to visualize the nuclear translocation of p65. As shown in Figure 4B, in unstimulated T98 cells, diffuse p65 staining was present, indicating that p65 could be distributed in the cytoplasm and nucleus simultaneously. In contrast, after 48 hours of 100 µM olanzapine treatment, most p65 was localized in the cytoplasm.

TNFα, a cytokine that can promote IκBα kinase (IKK) activity, targets IκBα degradation through the proteasome pathway, then activates NF‐κB and facilitates its translocation to the nucleus.^28^ Therefore, we used TNFα as a positive control for the activation of NF‐κB. As shown in Figure 4A, when TNFα (20 ng/mL, 2 hours) was used to stimulate T98 cells, NF‐κB in the cells was activated and translocated into the nucleus. However, compared with TNFα treatment alone, p65 protein level was decreased in the cytosol and increased in the nucleus with olanzapine treatment. Immunofluorescence assays indicated that p65 was mostly concentrated in the nucleus with TNFα treatment, while treatment with olanzapine caused p65 to leave from the nucleus to the cytoplasm (Figure 4B), indicating that olanzapine could substantially inhibit the nuclear localization of NF‐κB.

### 3.3 Olanzapine induced autophagy by inhibiting NF‐κB

**Figure 5:**
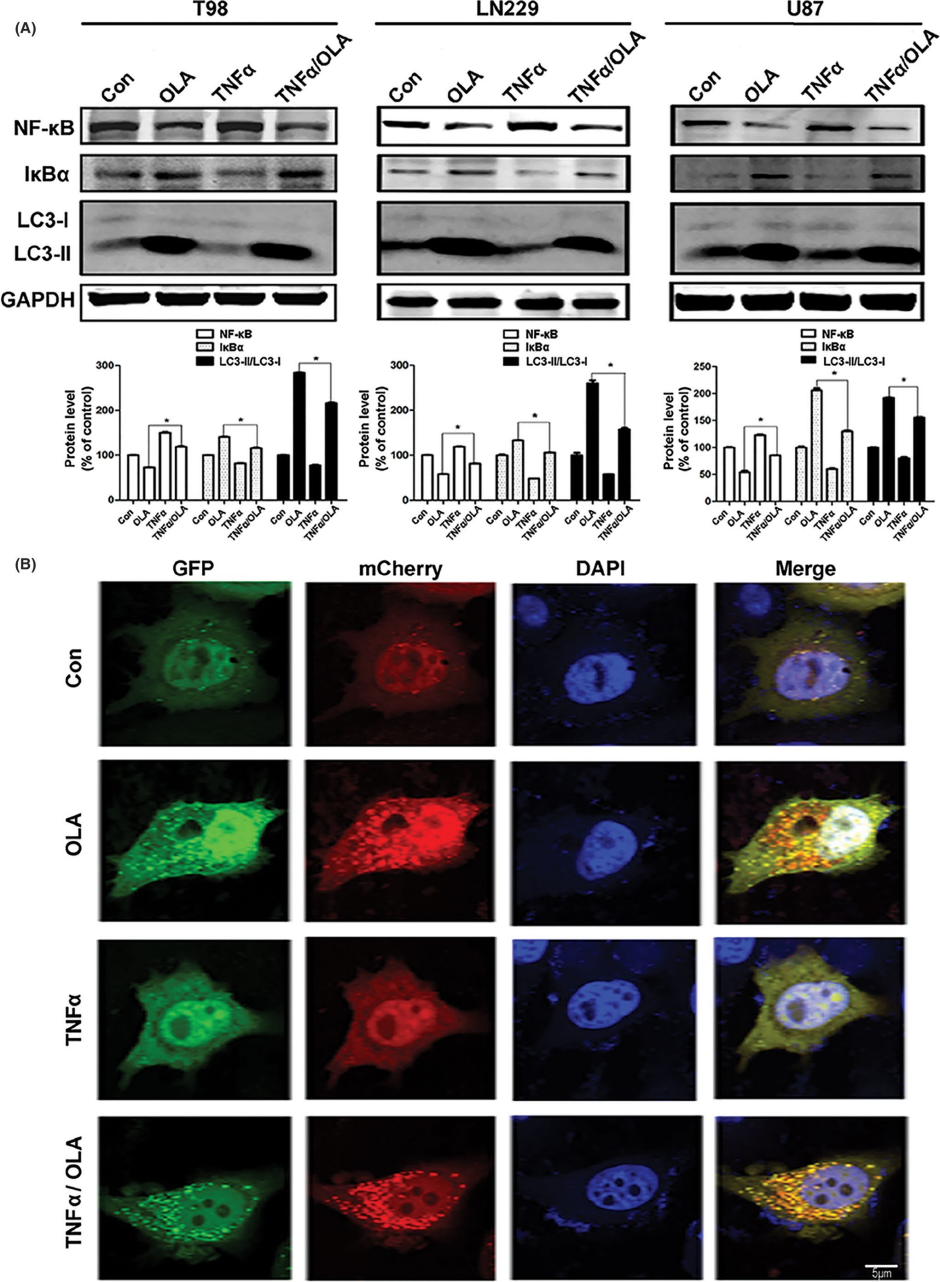
Suppression of NF‐κB activated autophagy with olanzapine treatment in glioma cells. A, T98 (100 μM), LN229 (100 μM), and U87 (50 μM) cells were subjected to OLA for 48 h with or without TNFa (20 ng/mL, 30 min) pretreatment. The NF‐κB, IκBa, and LC3 protein levels were analyzed by Western blotting (*P < 0.05 vs OLA group). B, Representative images of LC3 immunostaining were taken by confocal microscopy after OLA treatment (100 μM, 48 h) in T98 cells [Colour figure can be viewed at wileyonlinelibrary.com]

To further investigate whether NF‐κB inactivation plays a role in olanzapine‐induced autophagy in glioma cells, TNFα was used to alter the inhibitory effect of olanzapine on NF‐κB activity. As shown in Figure 5A, T98 cells pretreated with TNFα resulted in an inhibition of olanzapine‐induced autophagy compared with olanzapine treatment alone, indicating that olanzapine‐induced NF‐κB inhibition stimulates autophagy. The same mechanism was found to present in both LN229 and U87 glioma cell lines.

We then utilized GFP‐mCherry‐LC3 plasmids and examined the LC3 puncta to further verify the above findings. We observed more LC3 punctate in olanzapine‐treated T98 cells (100 µM, 48 hours), whereas a minimal amount of diffuse distribution of LC3 was observed in the untreated control group (Figure 5B). When treated with TNFα, autophagic flux was inhibited, but autophagy remained active with olanzapine cotreatment. Collectively, these data suggest that autophagic flux is activated by olanzapine in T98 cells. Thus, olanzapine may enhance anticancer effect by inhibiting NF‐κB and promoting increased autophagic cell death. In summary, these findings indicate that olanzapine treatment inhibits NF‐κB activity by preventing the transfer of the p65 subunit from the cellular cytoplasm into the nucleus, and this inhibition may be related to autophagy induction in glioma cells.

### 3.4 Olanzapine suppressed MGMT by inhibiting NF‐κB

**Figure 6:**
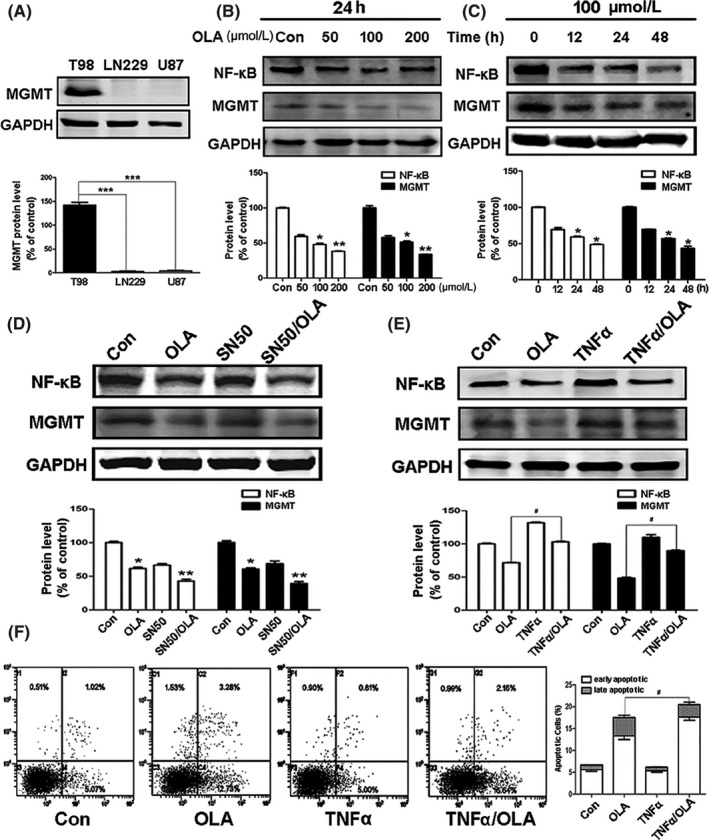
Olanzapine suppressed MGMT by inhibiting NF‐κB. (A) Total cell lysates were subjected to Western blot analysis to determine the protein levels of MGMT in T98, LN229, and U87cells (***P < 0.001 vs T98 cells). (B) T98 cells were treated with different concentrations for 24 h or (C) indicated concentration (100 μM) for different times, the NF‐κB and MGMT levels were evaluated by Western blotting (*P < 0.05, **P < 0.01 vs control). (D) T98 cells were subjected to 100 μM OLA for 48 h with or without SN50 (15 μM, 30 min) pretreatment. The NF‐κB and MGMT levels were analyzed by Western blotting (*P < 0.05; **P < 0.01; vs control). (E) T98 cells were subjected to OLA (100 µΜ, 48 h) with or without TNFa (20 ng/mL, 30 min) pretreatment. The NF‐κB and MGMT levels were evaluated by Western blotting (^#^ *P* < 0.05 vs OLA group). (F) Cells were treated with 100 μM OLA for 72 h with or without TNFa pretreatment. Annexin V‐FITC/PI staining was then used to determine the apoptosis in T98 cells (^#^ *P* < 0.05 vs OLA group)

T98 cells exhibit high level of MGMT expression, which is resistant to TMZ (Figure 6A). However, as shown in Figure 1A, olanzapine inhibited T98 cell activity, so we postulated that olanzapine could inhibit the expression of MGMT. As shown in Figure 6B,C, consistent with our hypothesis, olanzapine did have an inhibitory effect on MGMT in a dose and time‐dependent manner in T98 glioma cells. When olanzapine was used in combination with SN50 (NF‐κB inhibitor), the level of MGMT was significantly reduced (Figure 6D). In contrast, the activation of NF‐κB activity by TNFα increased the expression of MGMT (Figure 6E). In support of this observation, flow cytometry results revealed that the apoptosis coefficient was increased when cells were treated with olanzapine alone. This was not only related to olanzapine‐induced autophagy, but also may be related to the inhibition of MGMT, as MGMT inhibition could lead to autophagy induction and significantly increase autophagic cell death.^29^ In contrast, cotreatment with TNFα significantly decreased the apoptosis coefficient in T98 cell lines (Figure 6F). Our results indicate that olanzapine has important clinical value for MGMT‐expressing cells through the suppression of MGMT by NF‐κB inhibition.

## 4 DISCUSSION

The present study reports on a new antitumor mechanism of olanzapine. We found that olanzapine treatment had inhibitory effects on the proliferation of glioma cells through activation of autophagy (Figure 1). The autophagy marker LC3‐II accumulated, and autophagy vacuoles were also increased by olanzapine treatment. In addition, when combined with CQ and olanzapine, the LC3‐II form was significantly upregulated, and the inhibitory effects of olanzapine on glioma cells were enhanced. Several anti‐apoptotic genes, including Bcl‐2, were inhibited (Figure 2), suggesting that olanzapine may activate autophagic flux and promote autophagic cell death.

Cancer‐related autophagy often involves NF‐κB; however, reports of the relationship between olanzapine and NF‐κB are quite rare.^30^ Therefore, we studied the effect of olanzapine on NF‐κB and found that olanzapine could significantly inhibit its expression through repression of p65 nuclear translocation (Figure 4). P62, another autophagy marker protein which regulates NF‐κB signaling,^31^, ^32^ was also decreased by the inhibition of NF‐κB. However, when NF‐κB was activated by TNFα, it protected cells from autophagy and apoptosis.^33^

We also examined the role of LC3 by utilizing GFP‐mCherry‐LC3 plasmids. Following olanzapine treatment, NF‐κB decreased, LC3 increased, and autophagic flux was unobstructed. In contrast, after TNFα treatment, NF‐κB significantly increased while LC3 decreased and autophagic flux was inhibited. Finally, after TNFα pretreatment and subsequent olanzapine treatment, NF‐κB levels still decreased, LC3 increased, and autophagic flux changed from stagnation to patency (Figure 5). In conclusion, we confirmed that olanzapine was capable of activating autophagy and autophagic flux through the inhibition of NF‐κB.

MGMT‐expressing T98 cells are resistant to TMZ, and MGMT plays a key role in many types of cancers. Previous results indicate that NF‐κB is activated in response to alkylating agents and that high‐level activation of NF‐κB is associated with chemoresistance.^25^ In the present experiments, MGMT expression was positively correlated with NF‐κB activity. With olanzapine treatment, NF‐κB was inhibited and MGMT expression was decreased. In addition, olanzapine increased apoptosis and decreased proliferation (Figure 6). The results of SN50 treatment were similar to those of olanzapine treatment, and the greatest inhibitory effects were with combo of the two drugs. The results turned out oppositely when T98 cells were treated with TNFα, and MGMT expression was increased by NF‐κB activation (Figure 6E). The above results indicate that olanzapine could also inhibit T98 cell proliferation by blocking NF‐κB to inhibit MGMT.

Temozolomide is still the primary intervention used to treat glioma, and Karpel‐Massler et al believe that olanzapine combined with TMZ could enhance the antiproliferation effect of TMZ,^34^ so we attempted to treat glioma cell lines with TMZ in combination with olanzapine. However, the results were not satisfactory. We found that glioma cell viability with TMZ combined with olanzapine was higher than that of olanzapine alone. One potential explanation is that TMZ treatment activates NF‐κB,^35^ while olanzapine inhibits NF‐κB; therefore, the combo of TMZ and olanzapine resulted in lower efficacy than olanzapine treatment alone.

In conclusion, we demonstrated that olanzapine can induce autophagy by suppressing NF‐κB, thereby causing autophagic cell death and inhibiting glioma cell proliferation. Olanzapine can inhibit MGMT‐positive cell proliferation by inhibiting NF‐κB‐ and MGMT‐related activity. The identification of this pathway enhances our understanding on the mechanism of olanzapine's effects on tumor cell proliferation and provides a foundation for the development of olanzapine as a novel anticancer drug.

## CONFLICT OF INTEREST

The authors declare no conflict of interest, and all the authors listed have approved the manuscript.
